# The risk analysis index is an independent predictor of outcomes after lung cancer resection

**DOI:** 10.1371/journal.pone.0303281

**Published:** 2024-05-16

**Authors:** Andy Chao Hsuan Lee, Maria Lucia L. Madariaga, Sang Mee Lee, Mark K. Ferguson

**Affiliations:** 1 Section of Thoracic Surgery, Department of Surgery, University of Chicago, Chicago, Illinois, United States of America; 2 Department of Public Health Sciences, University of Chicago, Chicago, Illinois, United States of America; University of Torino, ITALY

## Abstract

**Background:**

The Risk Analysis Index (RAI) is a frailty assessment tool based on an accumulation of deficits model. We mapped RAI to data from the Society of Thoracic Surgeons (STS) Database to determine whether RAI correlates with postoperative outcomes following lung cancer resection.

**Methodology/Principal findings:**

This was a national database retrospective observational study based on data from the STS Database. Study patients underwent surgery 2018 to 2020. RAI was divided into four increasing risk categories. The associations between RAI and each of postoperative complications and administrative outcomes were examined using logistic regression models. We also compared the performance of RAI to established risk indices (American Society of Anesthesiology (ASA) and Charlson Comorbidity Index (CCI)) using areas under the Receiver Operating Characteristic (ROC) curves (AUC). Results: Of 29,420 candidate patients identified in the STS Database, RAI could be calculated for 22,848 (78%). Almost all outcome categories exhibited a progressive increase in marginal probability as RAI increased. On multivariable analyses, RAI was significantly associated with an incremental pattern with almost all outcomes. ROC analyses for RAI demonstrated “good” AUC values for mortality (0.785; 0.748) and discharge location (0.791), but only “fair” values for all other outcome categories (0.618 to 0.690). RAI performed similarly to ASA and CCI in terms of AUC score categories.

**Conclusions/Significance:**

RAI is associated with clinical and administrative outcomes following lung cancer resection. However, its overall accuracy as a surgical risk predictor is only moderate and similar to ASA and CCI. We do not recommend routine use of RAI for assessment of individual patient risk for major lung resection.

## Introduction

In 2022, there were 236,749 estimated number of new lung cancer cases and 130,180 estimated deaths from lung cancer [1]. As the projected number of people 65 years and older grows from 59 million in 2022 to 73 million in 2030 [2], thoracic surgeons are increasingly presented with the task of assessing operative candidacy and performing risk stratification for older patients with lung cancer. Chronological age does not always reflect biological age, and elderly people have a range of functional status that varies from robust to frail. Frailty has been identified as the progressive loss of physical and mental function that leads to an abnormal response to physiologic stressors [3]. In surgical fields, frailty correlates well with both mortality and morbidity across a broad spectrum of operations [4–6]. We recently characterized the 5-factor modified frailty index as predictive of most outcomes after major lung resection, but this metric was only marginally better than more commonly used risk assessment tools such as the American Society of Anesthesiology (ASA) Physical Status Classification System and Charlson Comorbidity Index (CCI) [7].

The original Risk Analysis Index was developed as a measure of frailty and used to predict 6-month mortality in nursing home residents [8]. This was later revised for surgical patients (Revised Risk Analysis Index, hereafter referred to as the Risk Analysis Index and abbreviated as RAI), and was externally validated using various national databases [9, 10]. Despite growing literature on RAI as a predictor of postoperative outcomes in high-risk operations including lung cancer resections [10, 11], there has not been any dedicated, in-depth analysis of RAI as a predictor of postoperative outcomes following major lung resection for cancer using the Society of Thoracic Surgery (STS) database. In the present study, we mapped the RAI variables to patient data from the STS General Thoracic Surgery Database and analyzed whether the RAI correlates with postoperative outcomes in patients following lung cancer resection.

## Materials and methods

### Data

The use of a limited dataset from the STS General Thoracic Surgery Database was approved by The University of Chicago Institutional Review Board (IRB21-0399, approved 3/9/2021) and the need for informed consent and consent documentation was waived. The STS General Thoracic Surgery Database contains more than 700,000 general thoracic surgery procedure records and currently has more than 1,000 participating surgeons [12]. We queried the STS database (Version 2.41) for all patients with a diagnosis of lung cancer undergoing elective anatomic lung resection from January 1, 2018 through December 31, 2020. Data were collected by the STS database during the period January 1, 2018 through June 30, 2021, and data were accessed July 26, 2021. Data were collected for demographic, physiologic, operative, and outcome variables. Comorbidities and ASA status were also collected. Patients were excluded if they underwent sleeve (carinal) pneumonectomy, extrapleural pneumonectomy, resection of an apical lung tumor including chest wall resection, completion pneumonectomy, chest wall reconstruction with muscle flap, or lung volume reduction surgery, or if they had a history of prior cardiothoracic surgery. Given that the patient data was de-identified, the authors had no access to identifying information during or after data collection. Because of purchase from a third party, the data cannot be made publicly available. Access to the data may be obtained through an application process (S1 Text).

### Outcome measures

Complications were categorized as pulmonary, cardiovascular, infectious, neurological, gastrointestinal, urinary, surgical, and in-hospital mortality (S1 Table). Composite outcomes included major postoperative complications and any postoperative event (S2 Table). Perioperative administrative outcomes included 30-day mortality, unexpected admission to the intensive care unit, readmission within 30 days of discharge, and discharge location other than home.

### Metrics

RAI was assessed by mapping ten variables in the STS database to eleven factors: age, sex, weight loss, poor appetite, congestive heart failure, dyspnea, renal failure, presence of cancer, functional status, cognitive decline, and living status (Table 1). An RAI score from 0 to 81 was calculated for each patient, with an increasing RAI score related to an increasing incidence of frailty. RAI was categorized into four subgroups (≤34, 35–39, 40–44, ≥45) as previously described [13]. Patients who did not have all the variables necessary for RAI calculation were included in a “missing” RAI category. The modified CCI was calculated as previously described [14]. ASA status was abstracted from STS data, which used a standard definition [15].

**Table 1:** Society of thoracic surgery database variables mapped to the Risk Analysis Index (RAI).

Risk Analysis Index variable	Matched variable(s) in STS Database Version 2.4	Risk Analysis Index score	
**Male sex**	Gender	3	
**Weight loss**	Unintentional weight loss in past three months (positive if > 10 lbs, or 4.5kg)	4	
**Poor appetite**	Unintentional weight loss in past three months (positive if > 10 lbs., or 4.5kg)	4	
**Renal failure**	On dialysis or last creatinine level (>1.86 mg/dl)	8	
**Chronic/congestive heart failure**	Congestive heart failure	5	
**Shortness of breath**	Category of Disease–Secondary (diagnosis 786.05, R06.02- Shortness of breath)	3	
**Residence other than individual living**	Living status	1	
**Cancer**	Lung cancer*	without cancer	with cancer
**Age**	Age at time of surgery		
**≤19**		0	28
**20–24**		1	29
**25–29**		4	29
**30–34**		6	30
**35–39**		8	30
**40–44**		10	31
**45–49**		12	31
**50–54**		14	32
**55–59**		16	32
**60–64**		18	33
**65–69**		20	34
**70–74**		22	34
**75–79**		24	35
**80–84**		26	35
**85–89**		28	36
**90–94**		30	36
**95–99**		32	37
**100+**		34	37
**Cognitive decline**	Dementia or neurocognitive dysfunction		
**Activities of daily living**	Functional status	Without cognitive decline	With cognitive decline
**Totally dependent**		14	16
**Partially dependent**		7	11
**Independent**		0	5

* All patients included in the study had diagnosis of lung cancer; unintentional weight loss was used twice in matching STS variables to RAI variables because no other STS variable was suitable for matching.

### Statistical techniques

Preoperative patient characteristics were compared among different RAI categories using Chi-square tests for categorical variables and ANOVA for continuous variables. Multivariable logistic regression models were used to evaluate the association between RAI categories and each of postoperative outcome controlling for extent of resection, induction therapy, body mass index (BMI) category, coronary artery disease (CAD), cerebrovascular disease, forced expiratory volume in the first second expressed as a percent of predicted (FEV1%), diffusing capacity of the lung for carbon monoxide expressed as a percent of predicted (DLCO%), hypertension, diabetes, and pathological T, N, and M stages [16] (S3 Table). Similar modeling was performed for CCI and ASA.

Predicted probability of each outcome for RAI risk categories was estimated from the multivariable logistic regression model. A progressive increase in the marginal probability as risk scores within a metric increased was interpreted as a desirable characteristic. Monotonicity was classified as strictly monotonic (progression between all ORs), partially monotonic (lack of progression between any two consecutive ORs), or non-monotonic. Receiver operating characteristic (ROC) analysis was performed for each instrument. The area under the ROC curve (AUC) for each pair of the instruments was compared using the DeLong test. All statistical analyses were performed using R software version 3.3.0.

## Results

### Patients

Of 29,420 patients identified in the STS General Thoracic Surgery Database according to the inclusion criteria and exclusion criteria, 22,848 (77.6%) had all the factors necessary for calculation of RAI (S1 Fig). Patients who lacked the necessary data to calculate RAI were most similar demographically and clinically to patients with an RAI score of 35–39 or 40–44, particularly with regards to age, gender, living status, shortness of breath, congestive heart failure, dialysis status, functional status, and neurocognitive dysfunction. These preoperative variables were included in the calculation of RAI and correlated positively with RAI (S4 Table). Among preoperative variables not included in the calculation of RAI, many comorbidities also had clinically significant correlations with RAI. There was no clinically significant change in distribution of BMI across RAI categories. Similarly, no consistent trends were seen with induction therapy with respect to RAI scores. However, patients with higher RAI scores were more likely to have higher pathological and clinical T stages for lung cancer. We compared the cohort that had the data needed for calculation of RAI to the cohort that did not and found no evidence of important clinical differences (S5 Table).

The median score for ASA and CCI was 3, whereas it was 36 for RAI (Table 2). The RAI and CCI scores were reasonably well distributed among three and four score categories, respectively, with sparse population of the one remaining category for RAI and among the three remaining relevant categories for CCI. Of note, CCI scores were always 2 or greater and RAI scores were always 28 or greater because all patients had a diagnosis of lung cancer. ASA was well distributed among three categories, with the remaining three categories being sparsely populated.

**Table 2:** Distribution of scores for the different risk assessment metrics among 29,420 patients.

Category	Metric		
	ASA	CCI	RAI
**Median**	3	3	36
**Score**	**Numbers of patients**		
0	n/a	0	n/a
1	70	0	n/a
2	4420	97	n/a
3	23075	15699	n/a
4	1845	7112	n/a
5	6	3617	n/a
6	4	1675	n/a
7	n/a	572	n/a
8–16	n/a	648	n/a
28–34	n/a	n/a	5991
35–39	n/a	n/a	14300
40–44	n/a	n/a	1774
> = 45	n/a	n/a	783

ASA: American Society of Anesthesiologists; CCI: Charlson Comorbidity Index; RAI: Revised Risk Analysis Index; 6,572 values missing

### Multivariate analyses

On multivariable analyses, RAI was significantly associated with an incremental pattern with almost all outcomes (Table 3). Multivariate analyses for ASA demonstrated a significant association with almost all postoperative and administrative outcomes with the exception of infectious complications, unexpected ICU admission, and unanticipated surgical approach conversion (S6 Table). CCI was significantly associated with almost all postoperative and administrative outcomes with the exception of infectious complications (S7 Table).

**Table 3:** Multivariate analysis for postoperative outcomes.

	Odds ratio relative to RAI ≤34							
	RAI 35–39	p-value	RAI 40–44	p-value	RAI ≥45	p-value	RAI Missing	p-value
**Postoperative complications**								
Pulmonary	1.18 (1.03, 1.36)	0.0190	1.40 (1.13, 1.72)	0.0017	1.73 (1.32, 2.24)	<0.0001	1.08 (0.92, 1.27)	0.3264
Cardiovascular	2.04 (1.80, 2.32)	<0.0001	2.01 (1.66, 2.42)	<0.0001	2.01 (1.56, 2.57)	<0.0001	1.62 (1.40, 1.87)	<0.0001
Infectious	1.23 (0.99, 1,54)	0.0625	1.16 (0.81, 1.64)	0.4103	1.95 (1.29, 2.88)	0.0011	1.27 (0.99, 1.63)	0.0578
Neurological	1.95 (1.50, 2.58)	<0.0001	2.84 (2.02, 4.01)	<0.0001	3.46 (2.30, 5.17)	<0.0001	2.19 (1.64, 2.94)	<0.0001
Gastrointestinal	1.42 (1.03, 1.99)	0.0362	1.70 (1.05, 2.70)	0.0278	2.82 (1.64, 4.71)	0.0001	1.69 (1.19, 2.43)	0.004
Urinary	1.81 (1.55, 2.13)	<0.0001	2.57 (2.07, 3.03)	<0.0001	3.03 (2.30, 3.95)	<0.0001	1.61 (1.35, 1.93)	<0.0001
Surgical	1.21 (1.11, 1.31)	<0.0001	1.26 (1.10, 1.45)	0.0012	1.39 (1.15, 1.68)	0.0005	1.02 (0.93, 1.13)	0.6490
**Administrative outcomes**								
In-hospital mortality	2.39 (1.31, 4.80)	0.0078	2.02 (0.85, 4.80)	0.1063	4.66 (1.99, 11.03)	0.0004	2.78 (1.46, 5.76)	0.0032
30-day mortality	2.39 (1.47, 4.16)	0.0010	3.44 (1.85, 6.55)	0.0001	5.08 (2.55, 10.17)	<0.0001	2.58 (1.52, 4.62)	0.0008
Unexpected ICU admission	1.48 (1.15, 1.93)	0.0031	1.73 (1.22, 2.45)	0.0022	1.44 (0.91, 2.23)	0.1061	1.50 (1.13, 2.02)	0.0063
Readmission within 30 days	1.24 (1.08, 1.43)	0.0019	1.72 (1.40, 2.10)	<0.0001	1.65 (1.24, 2.15)	0.0004	1.42(1.22, 1.65)	<0.0001
Unanticipated surgical approach conversion*	1.12 (0.98, 1.28)	0.1009	1.48 (1.21, 1.81)	0.0002	1.11 (0.81, 1.48)	0.5146	1.01 (0.86, 1.17)	0.9383
Discharge to home	0.61 (0.49, 0.74)	<0.0001	0.36 (0.28, 0.46)	<0.0001	0.19 (0.14, 0.25)	<0.0001	0.62 (0.50, 0.78)	<0.0001
**Composite events**								
Any post-operative event	1.46 (1.35, 1.57)	<0.0001	1.55 (1.37, 1.75)	<0.0001	1.90 (1.61, 2.25)	<0.0001	1.26 (1.16, 1.37)	<0.0001
Any major complication	1.47 (1.37, 1.58)	<0.0001	1.62 (1.44, 1.83)	<0.0001	1.99 (1.69, 2.36)	<0.0001	1.22 (1.12, 1.33)	<0.0001

RAI: Revised Risk Analysis Index; ICU: intensive care unit

*Video assisted thoracic surgery (VATS) to open or robotic to open

### Assessment of performance as risk metrics

Monotonicity of marginal probability was strict or partial among all outcome categories for RAI and ASA. Monotonicity of marginal probability was strict or partial for CCI among all outcome categories with the exception of gastrointestinal outcomes. The incidence of strict monotonicity was similar among CCI (11/15), RAI (9/15), and ASA (10/15). Of note, ASA was at less risk for partial monotonicity based on having only 3 categories compared to 4 for RAI and CCI (S8 Table).

ROC analyses demonstrated “good” AUC values (.70 to .80) for all three metrics related to mortality and discharge to other than home, whereas AUC values for other outcomes were in the fair category for all metrics (0.60 to 0.70; Table 4). Comparison of AUC values between each pair of the instruments using the DeLong test showed that RAI outperformed CCI and ASA in 6 of the 15 outcome categories, although the differences were not clinically important. There was no significant difference between CCI and ASA on ROC analyses (Table 4).

**Table 4:** Results of ROC analyses.

Complication/outcome category	AUC			p-values		
	RAI	ASA	CCI	RAI vs ASA	RAI vs CCI	ASA vs CCI
**Postoperative complications**						
Pulmonary	0.6766	0.6742	0.6743	0.1172	0.1198	0.9339
Cardiovascular	0.6234	0.6063	0.6047	< .0001	< .001	0.3881
Infectious	0.6241	0.6185	0.6192	0.0807	0.1552	0.7354
Neurological	0.6893	0.6811	0.6808	0.1290	0.0944	0.9355
Gastrointestinal	0.6403	0.6360	0.635	0.5157	0.4499	0.8615
Urinary	0.6221	0.6003	0.6029	< .0001	< .0001	0.2048
Surgical	0.6237	0.6225	0.6229	0.3059	0.5205	0.6628
In-hospital mortality	0.7850	0.7826	0.7799	0.7665	0.5354	0.6743
**Perioperative administrative outcomes**						
30-day mortality	0.7474	0.7343	0.7299	0.0951	0.0229	0.4016
Unexpected ICU admission	0.6517	0.6473	0.6501	0.2010	0.6763	0.3294
Readmission with 30 days	0.6112	0.6039	0.6037	0.0085	0.0067	0.8927
Discharge to home	0.7288	0.7128	0.7139	0.0001	0.0001	0.7113
Unanticipated surgical approach conversion*	0.6270	0.6254	0.6259	0.3646	0.5531	0.5886
**Composite events**						
Any post-operative events	0.6181	0.6118	0.6122	< .0001	0.0002	0.6749
Major complication	0.6233	0.6164	0.6166	< .0001	<0.0001	0.7885

ROC: receiver operating characteristics; AUC: area under the curve; RAI: Revised Risk Analysis Index; ASA: American Society of Anesthesiologists; CCI: Charlson Comorbidity Index; ICU: intensive care unit

* Video assisted thoracic surgery (VATS) to open or robotic to open

## Discussion

In many surgical fields, frailty has been shown to correlate with postoperative complications, discharge to institutional care, and mortality [17, 18]. The original Risk Analysis Index is a tool based on the accumulation of deficits model of frailty derived from the Minimum Set Mortality Risk Index-Revised (MMRI-R) instrument used to predict 6-month mortality in nursing home residents [8]. Arya et al. revised the original Risk Analysis Index scoring system for nonveteran surgical patients, externally validated the revised RAI using the National Surgical Quality Improvement Program Database (NSQIP) in patients undergoing elective noncardiac operations, and reported that RAI had improved discrimination and calibration as a frailty-screening tool in surgical patients compared to the original RAI [9]. In contrast, Wan et al. further investigated the accuracy of RAI in predicting postoperative morbidity and mortality in patients undergoing high-risk operations using NSQIP data and reported it to be an ineffective predictor of 30-day morbidity and mortality for patients undergoing high-risk operations including lung resection [11]. To date, no study has investigated the predictive value of the revised RAI (hereafter abbreviated as RAI) focusing on patients undergoing lung resection using the using the Society of Thoracic Surgery (STS) database. The current study mapped RAI variables to patient data from the STS database and assessed whether frailty as assessed by RAI is independently associated with postoperative outcomes in patients following anatomic lung resection for lung cancer.

We found RAI to be an independent predictor of all postoperative outcomes following anatomic lung section for lung cancer. On multivariable analysis, increasing RAI scores were associated with incremental changes in the odds of adverse outcomes that were clinically meaningful. We also demonstrated that RAI was associated with a wide variety of administrative outcomes. RAI performed similarly to ASA and CCI based on overall accuracy. Contrary to ROC analyses using the NSQIP database which identified RAI as a poor predictor of operative mortality [11], our analyses using the STS database suggested RAI to be a good predictor of postoperative mortality. However, RAI was similarly weak in predicting other postoperative outcomes compared to ASA and CCI, suggesting that its routine use in assessing surgical risk is only of moderate benefit.

An additional strength of RAI may be its potential ability to assess frailty. Tools assessing the “frailty phenotype” are sometimes impractical for routine screening in busy surgical clinics because they require time and effort from clinical staff to measure walking speed and grip strength [19]. RAI requires no special equipment and can be calculated in less than 60 seconds, allowing easy implementation and resulting in high participation compliance among surgical clinics [20]. However, although associations of RAI with surgical outcomes have been demonstrated, and its performance is similar to that of other frailty metrics generated using data from the electronic medical record, correlations between RAI and components of physical frailty such as weight loss, low gait speed, weakness, sarcopenia, and low energy have not been adequately assessed. In our study RAI did not correlate strongly with underweight status, suggesting a weak relationship with weight loss and weakness. Thus, whether RAI merely represents a potentially useful surgical risk tool or in addition has the added benefit of assessing frailty in a lung resection population has yet to be determined.

Although RAI may have the advantage of ease of calculation using the electronic medical record, its design as an accumulation of deficits model precludes extensive use of physical performance metrics for such calculation. Regardless of whether RAI is an accurate measure of frailty, the information from RAI cannot be used to identify individual elements in patients that might be targeted for mitigation to reduce operative risk, and RAI values for an individual patient will be unlikely to decrease in response to such mitigation. Therefore, RAI may serve primarily as a means for improved patient selection for surgery.

There are potential limitations to this study. Selecting covariates for multivariate analyses is sometimes challenging in studies such as this. There are a number of known factors that are associated with adverse clinical outcomes after resection for lung cancer that have been derived through assessment of the STS General Thoracic Surgery database [16]. Unfortunately, Version 2.41 of the STS data selected for the current study did not capture steroid use and Zubrod performance status, which have been shown to be positively correlated with postoperative mortality. Furthermore, gender and renal dysfunction could not be included in the multivariable model because they were included in the calculation of RAI. Review of the distribution of gender and renal dysfunction across the different RAI categories revealed strong collinearity between RAI and these variables (S4 Table). To directly compare the performance of ASA, CCI and RAI as surgical risk metrics, we removed ASA from the multivariable model when analyzing RAI and CCI.

There were a large number of missing values related to some outcome and covariate variables within the STS database. The general thoracic surgery STS database currently only receives data from 271 participant sites across North America, with center-level penetration between 40% and 50% [21], thus our findings may not be generalizable to all centers in North America. Data on post-discharge status are limited, largely restricting our analyses to in-hospital outcomes with no survival data. It is likely that a minimally invasive approach may lead to lower complication rates than an open approach, as is being studied in the ongoing VIOLET trial [22]. However, we did not assess surgical approach (open, VATS, robotic) and its possible effect on the utility of RAI in predicting complications, as this is the topic of a separate ongoing study using STS database data. Finally, we limited our study to Version 2.41 of the STS database due to changes in collected variables that permitted calculation of the RAI score using only this version.

We conclude that the RAI is associated with clinical and administrative outcomes following lung cancer resection. However, its overall accuracy as a surgical risk predictor is only moderate and similar to ASA and CCI. Our findings invite the question of whether RAI is a good frailty metric or is more accurately a surgical risk predictor in disguise. Future studies will be needed to investigate the relationship between RAI, physical frailty metrics, and sarcopenia.

## Supporting information

S1 Table Postoperative complication categories. (DOCX)

S2 Table Postoperative composite event definitions. (DOCX)

S3 Table Covariates for multivariate regression modeling. (DOCX)

S4 Table Preoperative demographic and clinical data including patients for whom RAI could not be calculated. (DOCX)

S5 Table Comparison between patients with and without RAI scores. (DOCX)

S6 Table Multivariate analysis for ASA and postoperative outcomes. (DOCX)

S7 Table Multivariate analysis for CCI and postoperative outcomes. (DOCX)

S8 Table Assessment of marginal probability for risk metrics by outcome categories. (DOCX)

S1 Fig Consort flow diagram. (TIF)

S1 Text (DOCX)
