# Supplementary material for: The risk analysis index is an independent predictor of outcomes after lung cancer resection
Source: PLoS One. 2024 May 16;19(5):e0303281. doi: 10.1371/journal.pone.0303281 (PMC11098335; doi:10.1371/journal.pone.0303281)
Supplement: S1 Table — (DOCX) [file pone.0303281.s001.docx]

**S1 Table. Postoperative complication categories**

| **Complication Category** | **Postoperative Event** |
| --- | --- |
| Pulmonary | Atelectasis requiring bronchoscopy, pneumonia, adult respiratory distress syndrome, initial ventilator support more than 48 hours, tracheostomy, respiratory failure, other pulmonary event |
| Cardiovascular | Deep venous thrombosis, atrial arrhythmia requiring treatment, ventricular arrhythmia requiring treatment, myocardial infarction, pulmonary embolism, other cardiovascular event |
| Infectious | Empyema, sepsis, surgical site infection, other infection requiring intravenous antibiotics |
| Neurological | Central neurological event, delirium, other neurological event |
| Gastrointestinal | Ileus, clostridium difficile infection, any other GI event |
| Urinary | New renal failure based on increase in serum creatinine level 3.0 x greater than baseline or new requirement for dialysis postoperatively, urinary tract infection, urinary retention, discharge with foley catheter |
| Surgical | Air leak greater than 5 days, intraoperative or perioperative blood transfusion, postoperative blood transfusion, chylothorax requiring medical therapy, pneumothorax requiring chest tube reinsertion, pleural effusion requiring drainage, unanticipated return to the OR, other events requiring OR with general anesthesia, bronchopleural fistula |
