# Supplementary material for: The risk analysis index is an independent predictor of outcomes after lung cancer resection
Source: PLoS One. 2024 May 16;19(5):e0303281. doi: 10.1371/journal.pone.0303281 (PMC11098335; doi:10.1371/journal.pone.0303281)
Supplement: S2 Table — (DOCX) [file pone.0303281.s002.docx]

**S2 Table. Postoperative composite event definitions**

| **Composite Event** | **Definition** |
| --- | --- |
| Major postoperative complication | Mortality during hospitalization, mortality within 30 days of the index operation, atelectasis requiring bronchoscopy, pneumonia, adult respiratory distress syndrome, initial ventilator support more than 48 hours, tracheostomy, respiratory failure, atrial arrhythmia requiring treatment, ventricular arrhythmia requiring treatment, myocardial infarction, pulmonary embolism, other major cardiovascular event, empyema, sepsis, surgical site infection, central neurological event, new renal failure, urinary tract infection, unanticipated return to the OR, other events requiring OR with general anesthesia, bronchopleural fistula, and readmission with 30 days of discharge |
| Any postoperative event | Any postoperative event that occurred at any time during hospital visit regardless of length of stay or within 30 days of surgery |
