# Supplementary material for: The risk analysis index is an independent predictor of outcomes after lung cancer resection
Source: PLoS One. 2024 May 16;19(5):e0303281. doi: 10.1371/journal.pone.0303281 (PMC11098335; doi:10.1371/journal.pone.0303281)
Supplement: S3 Table — (DOCX) [file pone.0303281.s003.docx]

**S3 Table. Covariates for multivariate regression modeling. Revised**

| **Covariate** | | |
| --- | --- | --- |
| Extent of resection | Lobectomy or segmentectomy | |
|  | Pneumonectomy | |
|  | Bilobectomy | |
|  |  | |
|  |  | |
| Induction therapy | No preoperative chemotherapy for the current thoracic malignancy AND prior radiation therapy to the chest for any reason | |
|  | Preoperative chemotherapy for the current thoracic malignancy AND prior radiation therapy to the chest for any reason | |
| Body mass index (BMI) category | Underweight (<18.5 kg/m^2^) |  |
|  | Normal (18.5 to 24.9 kg/m^2^) |  |
|  | Overweight (25 to 29.9 kg/m^2^) |  |
|  | Obese I (30 to 34.9 kg/m^2^) |  |
|  | Obese II (35 to 39.9 kg/m^2^) |  |
|  | Obese III (≥40 kg/m^2^) |  |
| Coronary artery disease (CAD) | | |
| Cerebrovascular disease (CVD) | No CVD |  |
|  | TIA |  |
|  | CVA |  |
| Forced expiratory volume in the first second expressed as a percent of predicted (FEV1%; 10 point increments) | | |
| Diffusing capacity of the lung for carbon monoxide expressed as a percent of predicted (DLCO%; 10 point increments) | | |
| Hypertension | | |
| Diabetes | | |
| Pathological stage | T status | T1 or T2 |
|  |  | T3 |
|  |  | T4 |
|  | N status | N0 |
|  |  | N1 |
|  |  | N2 or N3 |
|  | M status | M0 |
|  |  | M1 |
