# Supplementary material for: The risk analysis index is an independent predictor of outcomes after lung cancer resection
Source: PLoS One. 2024 May 16;19(5):e0303281. doi: 10.1371/journal.pone.0303281 (PMC11098335; doi:10.1371/journal.pone.0303281)
Supplement: S4 Table — (DOCX) [file pone.0303281.s004.docx]

**S4 Table. Preoperative demographic and clinical data including patients for whom RAI could not be calculated.**

| **Characteristic** |  | **RAI** | | | | | | | | | | | |  |
| --- | --- | --- | --- | --- | --- | --- | --- | --- | --- | --- | --- | --- | --- | --- |
|  |  | **<=34**  **n=5991** | | **35-39**  **n=14300** | | **40-44**  **n=1774** | | **>=45**  **n=783** | | **Missing**  **n=6572** | | **Total**  **n=29,420** | | **p-value** |
|  |  | **n** | **%** | **n** | **%** | **n** | **%** | **n** | **%** | **n** | **%** | **n** | **%** |  |
| **Age*** | **<=19** | 38 | 0.63 | 4 | 0.03 | 2 | 0.11 | 0 | 0 | 15 | 0.23 | 59 | 0.20 | <0.0001 |
|  | **20-24** | 28 | 0.47 | 1 | 0.01 | 1 | 0.06 | 0 | 0 | 7 | 0.11 | 37 | 0.13 |  |
|  | **25-29** | 35 | 0.58 | 0 | 0 | 0 | 0 | 0 | 0 | 22 | 0.33 | 57 | 0.19 |  |
|  | **30-34** | 70 | 1.17 | 0 | 0 | 3 | 0.17 | 0 | 0 | 23 | 0.35 | 96 | 0.33 |  |
|  | **35-39** | 105 | 1.75 | 3 | 0.02 | 5 | 0.28 | 0 | 0 | 36 | 0.55 | 149 | 0.51 |  |
|  | **40-44** | 137 | 2.29 | 59 | 0.41 | 10 | 0.56 | 0 | 0 | 59 | 0.9 | 265 | 0.90 |  |
|  | **45-49** | 276 | 4.61 | 126 | 0.88 | 28 | 1.58 | 2 | 0.26 | 126 | 1.92 | 558 | 1.90 |  |
|  | **50-54** | 559 | 9.33 | 334 | 2.34 | 69 | 3.89 | 3 | 0.38 | 289 | 4.4 | 1254 | 4.26 |  |
|  | **55-59** | 1353 | 22.58 | 892 | 6.24 | 186 | 10.48 | 27 | 3.45 | 683 | 10.39 | 3141 | 10.68 |  |
|  | **60-64** | 1985 | 33.13 | 1403 | 9.81 | 222 | 12.51 | 88 | 11.24 | 1057 | 16.08 | 4755 | 16.16 |  |
|  | **65-69** | 648 | 10.82 | 3656 | 25.57 | 282 | 15.9 | 179 | 22.86 | 1384 | 21.06 | 6149 | 20.90 |  |
|  | **70-74** | 757 | 12.64 | 3486 | 24.38 | 371 | 20.91 | 200 | 25.54 | 1435 | 21.84 | 6249 | 21.24 |  |
|  | **75-79** | 0 | 0 | 2974 | 20.8 | 332 | 18.71 | 164 | 20.95 | 974 | 14.82 | 4444 | 15.11 |  |
|  | **80-84** | 0 | 0 | 1196 | 8.36 | 134 | 7.55 | 88 | 11.24 | 363 | 5.52 | 1781 | 6.05 |  |
|  | **85-89** | 0 | 0 | 152 | 1.06 | 121 | 6.82 | 30 | 3.83 | 91 | 1.38 | 394 | 1.34 |  |
|  | **90-95** | 0 | 0 | 14 | 0.1 | 7 | 0.39 | 2 | 0.26 | 7 | 0.11 | 30 | 0.10 |  |
|  | **100+** | 0 | 0 | 0 | 0 | 1 | 0.06 | 0 | 0 | 1 | 0.02 | 2 | 0.01 |  |
| **Sex*** | **Male** | 149 | 2.49 | 8020 | 56.08 | 756 | 42.62 | 659 | 84.16 | 2735 | 41.62 | 12319 | 41.87 | <0.0001 |
|  | **Female** | 5842 | 97.51 | 6280 | 43.92 | 1018 | 57.38 | 124 | 15.84 | 3834 | 58.34 | 17098 | 58.12 |  |
| **Living Status*** | **Alone** | 2391 | 39.91 | 2539 | 17.76 | 409 | 23.06 | 117 | 14.94 | 1231 | 18.73 | 6687 | 22.73 | <0.0001 |
|  | **With Family or Friend** | 3596 | 60.02 | 11732 | 82.04 | 1340 | 75.54 | 631 | 80.59 | 3920 | 59.65 | 21219 | 72.12 |  |
|  | **Assisted Living** | 2 | 0.03 | 25 | 0.17 | 14 | 0.79 | 23 | 2.94 | 18 | 0.27 | 82 | 0.28 |  |
|  | **Nursing Home** | 2 | 0.03 | 4 | 0.03 | 11 | 0.62 | 12 | 1.53 | 5 | 0.08 | 34 | 0.12 |  |
| **Shortness of Breath*** | | 0 | 0 | 75 | 0.52 | 43 | 2.42 | 19 | 2.43 | 23 | 0.35 | 160 | 0.54 | <0.0001 |
| **Congestive Heart Failure*** | | 2 | 0.03 | 106 | 0.74 | 535 | 30.16 | 135 | 17.24 | 207 | 3.15 | 985 | 3.35 | 0.0000 |
| **Dialysis *** | | 0 | 0 | 2 | 0.01 | 34 | 1.92 | 33 | 4.21 | 27 | 0.41 | 96 | 0.33 | 0.0000 |
| **Renal dysfunction** | **No** | 5582 | 93 | 13479 | 94 | 1613 | 91 | 621 | 79 | 5917 | 90 | 25712 | 92 | <0.0001 |
|  | **Yes** | 409 | 7 | 821 | 6 | 161 | 9 | 162 | 21 | 655 | 10 | 2208 | 8 |  |
| **Functional Status*** | **Independent** | 5991 | 100 | 14290 | 99.93 | 1417 | 79.88 | 511 | 65.26 | 5219 | 79.41 | 27428 | 93.23 | <0.0001 |
|  | **Partially Dependent** | 0 | 0 | 10 | 0.07 | 357 | 20.12 | 258 | 32.95 | 115 | 1.75 | 740 | 2.52 |  |
|  | **Totally Dependent** | 0 | 0 | 0 | 0 | 0 | 0 | 14 | 1.79 | 1 | 0.02 | 15 | 0.05 |  |
|  | **Unknown** | 0 | 0 | 0 | 0 | 0 | 0 | 0 | 0 | 41 | 0.62 | 41 | 0.14 |  |
| **Dementia or Neurocognitive Dysfunction*** | | 1 | 0.02 | 38 | 0.27 | 177 | 9.98 | 93 | 11.88 | 57 | 0.87 | 366 | 1.24 | <0.0001 |
| **Race** | **Caucasian** | 4881 | 81.47 | 12190 | 85.24 | 1445 | 81.45 | 643 | 82.12 | 5490 | 83.54 | 24649 | 83.78 | <0.0001 |
|  | **Black** | 656 | 10.95 | 1035 | 7.24 | 220 | 12.4 | 98 | 12.52 | 514 | 7.82 | 2523 | 8.58 | <0.0001 |
|  | **Asian** | 247 | 4.12 | 604 | 4.22 | 46 | 2.59 | 20 | 2.55 | 243 | 3.7 | 1160 | 3.94 | <0.0001 |
|  | **Native American** | 21 | 0.35 | 55 | 0.38 | 10 | 0.56 | 4 | 0.51 | 20 | 0.3 | 110 | 0.37 | <0.0001 |
|  | **Race Other** | 135 | 2.25 | 305 | 2.13 | 51 | 2.87 | 19 | 2.43 | 128 | 1.95 | 638 | 2.17 | <0.0001 |
|  | **Hispanic** | 218 | 3.64 | 510 | 3.57 | 76 | 4.28 | 28 | 3.58 | 189 | 2.88 | 1021 | 3.47 | <0.0001 |
| **Body Mass Index (kg/m^2^)** | **<18.5** | 196 | 3.27 | 301 | 2.1 | 75 | 4.23 | 21 | 2.68 | 173 | 2.63 | 766 | 2.60 | <0.0001 |
|  | **18.5-25** | 2013 | 33.6 | 4444 | 31.08 | 512 | 28.86 | 252 | 32.18 | 2120 | 32.26 | 9341 | 31.75 |  |
|  | **25-30** | 1801 | 30.06 | 5291 | 36.37 | 596 | 33.6 | 265 | 33.84 | 2274 | 34.6 | 10227 | 34.76 |  |
|  | **30-35** | 1119 | 18.68 | 2858 | 19.99 | 359 | 20.24 | 170 | 21.71 | 1230 | 18.72 | 5736 | 19.50 |  |
|  | **35-40** | 547 | 9.13 | 1025 | 7.17 | 160 | 9.02 | 59 | 7.54 | 522 | 7.94 | 2313 | 7.86 |  |
|  | **≥40** | 314 | 5.24 | 379 | 2.65 | 72 | 4.06 | 16 | 2.04 | 223 | 3.39 | 1004 | 3.41 |  |
| **Cigarette Smoking** | **Never** | 1489 | 24.85 | 2499 | 17.48 | 275 | 15.5 | 64 | 8.17 | 1296 | 19.72 | 5623 | 19.11 | <0.0001 |
|  | **Past Smoker** | 2881 | 48.09 | 8684 | 60.73 | 1051 | 59.24 | 514 | 65.64 | 3846 | 58.52 | 16976 | 57.70 |  |
|  | **Current Smoker** | 1619 | 27.02 | 3113 | 21.77 | 447 | 25.2 | 205 | 26.18 | 1429 | 21.74 | 6813 | 23.16 |  |
| **Pulmonary Hypertension** | | 60 | 1 | 229 | 1.6 | 68 | 3.83 | 27 | 3.45 | 104 | 1.58 | 488 | 1.66 | <0.0001 |
| **Interstitial Fibrosis** | | 67 | 1.12 | 227 | 1.59 | 50 | 2.82 | 24 | 3.07 | 81 | 1.23 | 449 | 1.53 | <0.0001 |
| **Hypertension** | | 2883 | 48.12 | 9719 | 67.97 | 1352 | 76.21 | 627 | 80.08 | 4053 | 61.67 | 18634 | 63.34 | <0.0001 |
| **ECOG Score** | **0 or 1** | 5854 | 97.71 | 13831 | 96.72 | 1557 | 87.77 | 636 | 81.23 | 6316 | 96.1 | 28194 | 95.83 | <0.0001 |
|  | **2** | 95 | 1.59 | 350 | 2.45 | 173 | 9.75 | 107 | 13.67 | 177 | 2.69 | 902 | 3.07 |  |
|  | **≥3** | 5 | 0.08 | 37 | 0.26 | 34 | 1.92 | 35 | 4.47 | 25 | 0.38 | 136 | 0.46 |  |
| **Coronary Artery Disease** | | 473 | 7.9 | 2649 | 18.52 | 545 | 30.72 | 252 | 32.18 | 990 | 15.06 | 4909 | 16.69 | <0.0001 |
| **History of Myocardial Infarction** | | 190 | 3.17 | 873 | 6.1 | 219 | 12.34 | 101 | 12.9 | 393 | 5.98 | 1776 | 6.04 | <0.0001 |
| **Atrial Fibrillation** | | 130 | 2.17 | 1096 | 7.66 | 260 | 14.66 | 106 | 13.54 | 428 | 6.51 | 2020 | 6.87 | <0.0001 |
| **Valvular Heart Disease** | | 148 | 2.47 | 532 | 3.72 | 128 | 7.22 | 53 | 6.77 | 200 | 3.04 | 1061 | 3.61 | <0.0001 |
| **Diabetes** | | 876 | 14.62 | 2973 | 20.79 | 528 | 29.76 | 276 | 35.25 | 1207 | 18.37 | 5860 | 19.92 | <0.0001 |
| **Major Vascular Disease** | | 286 | 4.77 | 1416 | 9.9 | 260 | 14.66 | 139 | 17.75 | 510 | 7.76 | 2611 | 8.87 | <0.0001 |
| **Cerebrovascular Disease History** | **None** | 5605 | 93.56 | 12978 | 90.76 | 1501 | 84.61 | 640 | 81.74 | 5967 | 90.79 | 26691 | 90.72 | <0.0001 |
|  | **TIA** | 131 | 2.19 | 454 | 3.17 | 78 | 4.4 | 30 | 3.83 | 179 | 2.72 | 872 | 2.96 |  |
|  | **CVA** | 195 | 3.25 | 611 | 4.27 | 156 | 8.79 | 91 | 11.62 | 285 | 4.34 | 1338 | 4.55 |  |
|  | **Known disease, no events** | 55 | 0.92 | 241 | 1.69 | 38 | 2.14 | 20 | 2.55 | 80 | 1.22 | 434 | 1.48 |  |
| **Permanent Neurologic Impairment** | | 43 | 0.72 | 156 | 1.09 | 60 | 3.38 | 39 | 4.98 | 82 | 1.25 | 380 | 1.29 | <0.0001 |
| **Liver Dysfunction** | | 114 | 1.9 | 321 | 2.24 | 71 | 4 | 32 | 4.09 | 139 | 2.12 | 677 | 2.30 | <0.0001 |
| **Preoperative Radiation Therapy** | | 423 | 7.06 | 802 | 5.61 | 114 | 6.43 | 45 | 5.75 | 339 | 5.16 | 1723 | 5.86 | <0.0001 |
| **Preoperative Chemotherapy** | | 649 | 10.83 | 1342 | 9.38 | 177 | 9.98 | 90 | 11.49 | 646 | 9.83 | 2904 | 9.87 | <0.0001 |
| **Clinical Staging T** | **Tis, T1** | 4097 | 68.39 | 9290 | 64.97 | 1057 | 59.58 | 450 | 57.47 | 4203 | 63.95 | 19097 | 64.91 | <0.0001 |
|  | **T2** | 1113 | 18.58 | 2954 | 20.66 | 443 | 24.97 | 195 | 24.9 | 1324 | 20.15 | 6029 | 20.49 |  |
|  | **T3** | 392 | 6.54 | 1091 | 7.63 | 137 | 7.72 | 76 | 9.71 | 514 | 7.82 | 2210 | 7.51 |  |
|  | **T4** | 136 | 2.27 | 412 | 2.88 | 65 | 3.66 | 37 | 4.73 | 217 | 3.3 | 867 | 2.95 |  |
| **Clinical Staging N** | **N0** | 5215 | 87.05 | 12439 | 86.99 | 1517 | 85.51 | 677 | 86.46 | 5625 | 85.59 | 25473 | 86.58 | 0.0183 |
|  | **N1** | 323 | 5.39 | 842 | 5.89 | 131 | 7.38 | 54 | 6.9 | 381 | 5.8 | 1731 | 5.88 |  |
|  | **N2** | 210 | 3.51 | 468 | 3.27 | 57 | 3.21 | 27 | 3.45 | 253 | 3.85 | 1015 | 3.45 |  |
|  | **N3** | 13 | 0.22 | 25 | 0.17 | 5 | 0.28 | 0 | 0 | 17 | 0.26 | 60 | 0.20 |  |
| **Clinical Staging M** | **M0** | 5693 | 95.03 | 13653 | 95.48 | 1686 | 95.04 | 749 | 95.66 | 6206 | 94.43 | 27987 | 95.13 | 0.0251 |
|  | **M1** | 70 | 1.17 | 125 | 0.87 | 24 | 1.35 | 9 | 1.15 | 68 | 1.03 | 296 | 1.01 |  |
| **Pathological Staging T** | **T1** | 3647 | 60.87 | 7770 | 54.34 | 873 | 49.21 | 346 | 44.19 | 3675 | 55.92 | 16311 | 55.44 | <0.0001 |
|  | **T2** | 1621 | 27.06 | 4366 | 30.53 | 572 | 32.24 | 261 | 33.33 | 1893 | 28.8 | 8713 | 29.62 |  |
|  | **T3** | 493 | 8.23 | 1506 | 10.53 | 215 | 12.12 | 117 | 14.94 | 679 | 10.33 | 3010 | 10.23 |  |
|  | **T4** | 173 | 2.89 | 550 | 3.85 | 93 | 5.24 | 50 | 6.39 | 274 | 4.17 | 1140 | 3.87 |  |
| **Pathological Staging N** | **N0** | 4770 | 79.62 | 11237 | 78.58 | 1342 | 75.65 | 608 | 77.65 | 5169 | 78.65 | 23126 | 78.61 | 0.0029 |
|  | **N1** | 679 | 11.33 | 1806 | 12.63 | 253 | 14.26 | 102 | 13.03 | 799 | 12.16 | 3639 | 12.37 |  |
|  | **N2** | 446 | 7.44 | 1043 | 7.29 | 136 | 7.67 | 55 | 7.02 | 519 | 7.9 | 2199 | 7.47 |  |
|  | **N3** | 4 | 0.07 | 4 | 0.03 | 1 | 0.06 | 0 | 0 | 1 | 0.02 | 10 | 0.03 |  |
|  | **NX** | 38 | 0.63 | 107 | 0.75 | 21 | 1.18 | 9 | 1.15 | 29 | 0.44 | 204 | 0.69 |  |
| **Pathological Staging M** | **M0** | 5861 | 97.83 | 14020 | 98.04 | 1722 | 97.07 | 761 | 97.19 | 6420 | 97.69 | 28784 | 97.84 | 0.1382 |
|  | **M1** | 70 | 1.17 | 167 | 1.17 | 27 | 1.52 | 12 | 1.53 | 83 | 1.26 | 359 | 1.22 |  |
|  |  | **RAI** | | | | | | | | | | | |  |
|  |  | **<=34**  **n=5991** | | **35-39**  **n=14300** | | **40-44**  **n=1774** | | **>=45**  **n=783** | | **Missing**  **n=6572** | | **Total**  **n=29,420** | | **p-value** |
| **FEV1% (mean ± SD)** | | 87.1 **±** 18.7 | | 86.5 **±** 20.0 | | 83.4 **±** 20.2 | | 81.3 **±** 18.5 | | 87.2 **±** 19.9 | | 86.4 **±** 19.8 | | <0.0001 |
| **DLCO% (mean ± SD)** | | 77.7 **±** 20.1 | | 77.5 **±** 20.9 | | 72.2 **±** 20.9 | | 71.8 **±** 20.1 | | 77.6 **±** 21.0 | | 77.1 **±** 20.8 | | <0.0001 |
| **Pack Years (mean ± SD)** | | 35.5 **±** 22.7 | | 42.8 **±** 27.6 | | 42.8 **±** 28.4 | | 51.9 **±** 33.2 | | 39.9 **±** 25.9 | | 41.1 **±** 26.8 | | <0.0001 |

*Variables involved in calculation of RAI scores; CVA: cerebrovascular accident; ECOG Score: Eastern Cooperative Oncology Group Performance Status Score; FEV1%: forced expiratory volume during the first second expressed as a percent of predicted; RAI: Risk Analysis Index; SD: Standard deviation; TIA: transient ischemic attack; DLCO%: diffusing capacity for carbon monoxide expressed as a percent of predicted
