# Supplementary material for: The risk analysis index is an independent predictor of outcomes after lung cancer resection
Source: PLoS One. 2024 May 16;19(5):e0303281. doi: 10.1371/journal.pone.0303281 (PMC11098335; doi:10.1371/journal.pone.0303281)
Supplement: S5 Table — (DOCX) [file pone.0303281.s005.docx]

**S5 Table. Comparison between patients with and without RAI scores**

| **Characteristic** | | **RAI available** | | **RAI not available** | | **Total** | |  |
| --- | --- | --- | --- | --- | --- | --- | --- | --- |
|  |  | **n** | **%** | **n** | **%** | **n** | **%** | **p-value** |
| **Race** | **Caucasian** | 19156 | 83.9 | 5493 | 83.5 | 24649 | 83.8 | 0.008 |
|  | **Black** | 2009 | 8.79 | 514 | 7.82 | 2523 | 8.58 | <0.001 |
|  | **Asian** | 917 | 4.01 | 243 | 3.69 | 1160 | 3.94 | <0.001 |
|  | **Native American** | 90 | 0.39 | 20 | 0.30 | 110 | 0.37 | <0.001 |
|  | **Race Other** | 509 | 2.23 | 129 | 1.96 | 638 | 2.17 | <0.001 |
|  | **Hispanic** | 831 | 3.64 | 190 | 2.89 | 1021 | 3.47 | <0.001 |
| **Body Mass Index (kg/m^2^)** | **<18.5** | 593 | 2.6 | 173 | 2.63 | 766 | 2.60 | 0.651 |
|  | **18.5 to 24.9** | 7219 | 31.6 | 2122 | 32.3 | 9341 | 31.8 |  |
|  | **25 to 29.9** | 7952 | 34.8 | 2275 | 34.6 | 10227 | 34.8 |  |
|  | **30 to 34.9** | 4504 | 19.7 | 1232 | 18.7 | 5736 | 19.5 |  |
|  | **35 to 39.9** | 1791 | 7.84 | 522 | 7.94 | 2313 | 7.86 |  |
|  | **≥40** | 781 | 3.42 | 223 | 3.39 | 1004 | 3.41 |  |
| **Cigarette Smoking** | **Never** | 4326 | 18.9 | 1297 | 19.7 | 5623 | 19.1 | 0.014 |
|  | **Past Smoker** | 13126 | 57.5 | 3850 | 58.5 | 16976 | 57.7 |  |
|  | **Current Smoker** | 5384 | 23.6 | 1429 | 21.7 | 6813 | 23.2 |  |
| **Pulmonary Hypertension** | | 384 | 1.68 | 104 | 1.58 | 488 | 1.66 | <0.001 |
| **Interstitial Fibrosis** | | 368 | 1.61 | 81 | 1.23 | 449 | 1.53 | 0.016 |
| **Hypertension** | | 14579 | 63.8 | 4055 | 61.7 | 18634 | 63.3 | <0.001 |
| **ECOG Score** | **0 or 1** | 21873 | 95.8 | 6321 | 96.1 | 28194 | 95.8 | 0.076 |
|  | **2** | 725 | 3.17 | 177 | 2.69 | 902 | 3.07 |  |
|  | **≥3** | 111 | 0.49 | 25 | 0.38 | 136 | 0.46 |  |
| **Coronary Artery Disease** | | 3918 | 17.1 | 991 | 15.1 | 4909 | 16.7 | <0.001 |
| **History of Myocardial Infarction** | | 1382 | 6.05 | 394 | 5.99 | 1776 | 6.04 | <0.001 |
| **Atrial Fibrillation** | | 1591 | 6.96 | 429 | 6.52 | 2020 | 6.87 | <0.001 |
| **Valvular Heart Disease** | | 861 | 3.77 | 200 | 3.04 | 1061 | 3.61 | <0.001 |
| **Diabetes** | | 4652 | 20.4 | 1208 | 18.4 | 5860 | 19.9 | <0.001 |
| **Major Vascular Disease** | | 2100 | 9.19 | 511 | 7.77 | 2611 | 8.87 | <0.001 |
| **Cerebrovascular Disease History** | **None** | 20719 | 90.7 | 5972 | 90.8 | 26691 | 90.7 | <0.001 |
|  | **TIA** | 693 | 3.03 | 179 | 2.72 | 872 | 2.96 |  |
|  | **CVA** | 1053 | 4.61 | 285 | 4.33 | 1338 | 4.55 |  |
|  | **Known disease, no event** | 354 | 1.55 | 80 | 1.22 | 434 | 1.48 |  |
| **Permanent Neurologic Impairment** | | 298 | 1.30 | 82 | 1.25 | 380 | 1.29 | <0.001 |
| **Liver Dysfunction** | | 538 | 2.36 | 139 | 2.11 | 677 | 2.30 | <0.001 |
| **Preoperative Radiation Therapy** | | 1384 | 6.06 | 339 | 5.15 | 1723 | 5.86 | <0.001 |
| **Preoperative Chemotherapy** | | 2258 | 9.88 | 646 | 9.82 | 2904 | 9.8 | <0.001 |
| **Clinical Staging T** | **Tis, T1** | 14891 | 65.2 | 4206 | 64.0. | 19097 | 64.9 | 0.004 |
|  | **T2** | 4705 | 20.6 | 1324 | 20.1 | 6029 | 20.5 |  |
|  | **T3** | 1695 | 7.42 | 515 | 7.83 | 2210 | 7.51 |  |
|  | **T4** | 649 | 2.84 | 218 | 3.31 | 867 | 2.95 |  |
| **Clinical Staging N** | **N0** | 19843 | 869 | 5630 | 85.6 | 25473 | 86.6 | 0.006 |
|  | **N1** | 1350 | 5.91 | 381 | 5.79 | 1731 | 5.88 |  |
|  | **N2** | 762 | 3.34 | 253 | 3.85 | 1015 | 3.45 |  |
|  | **N3** | 43 | 0.19 | 17 | 0.26 | 60 | 0.20 |  |
| **Clinical Staging M** | **M0** | 21776 | 95.3 | 6211 | 94.4 | 27987 | 95.1 | 0.748 |
|  | **M1** | 228 | 1.04 | 68 | 1.03 | 296 | 1.01 |  |
| **Pathological Staging T** | **T1** | 12633 | 55.3 | 3678 | 55.9 | 16311 | 55.4 | 0.214 |
|  | **T2** | 6820 | 29.9 | 1893 | 28.8 | 8713 | 29.6 |  |
|  | **T3** | 2330 | 10.2 | 680 | 10.3 | 3010 | 10.2 |  |
|  | **T4** | 865 | 3.79 | 275 | 4.18 | 1140 | 3.87 |  |
| **Pathological Staging N** | **N0** | 17953 | 78.6 | 5173 | 78.7 | 23126 | 78.6 | 0.073 |
|  | **N1** | 2840 | 12.4 | 799 | 12.2 | 3639 | 12.4 |  |
|  | **N2** | 1680 | 7.35 | 519 | 7.89 | 2199 | 7.47 |  |
|  | **N3** | 9 | 0.04 | 1 | 0.02 | 10 | 0.03 |  |
|  | **NX** | 174 | 0.76 | 30 | 0.46 | 204 | 0.69 |  |
| **Pathological Staging M** | **M0** | 22359 | 97.9 | 6425 | 97.7 | 28784 | 97.8 | 0.554 |
|  | **M1** | 276 | 1.21 | 83 | 1.26 | 359 | 1.22 |  |
|  |  | **RAI available** | | **RAI not available** | | **Total** | | **p-value** |
| **FEV1% (mean ± SD)** |  | 86.2 **±** 19.74 | | 87.2 **±** 19.9 | | 86.4 **±** 19.8 | | <0.001 |
| **DLCO% (mean ± SD)** |  | 76.9 **±** 20.7 | | 77.6 **±** 21.0 | | 77.1 **±** 20.8 | | 0.025 |
| **Pack Years (mean ± SD)** |  | 41.4 **±** 27.1 | | 39.9 **±** 25.9 | | 41.1 **±** 26.8 | | <0.001 |

CVA: cerebrovascular accident; ECOG Score: Eastern Cooperative Oncology Group Performance Status Score; FEV1%: forced expiratory volume during the first second expressed as a percent of predicted; RAI: Risk Analysis Index; SD: Standard Deviation; TIA: transient ischemic attack; DLCO%: diffusing capacity for carbon monoxide expressed as a percent of predicted
