# Supplementary material for: The risk analysis index is an independent predictor of outcomes after lung cancer resection
Source: PLoS One. 2024 May 16;19(5):e0303281. doi: 10.1371/journal.pone.0303281 (PMC11098335; doi:10.1371/journal.pone.0303281)
Supplement: S6 Table — (DOCX) [file pone.0303281.s006.docx]

**S6 Table. Multivariate analysis for ASA and postoperative outcomes**

|  | **Odds ratio* (95% confidence interval)** | | | |
| --- | --- | --- | --- | --- |
|  | ASA = 3 | p-value | ASA = 4,5,6 | p-value |
| **Postoperative complications** | | | | |
| Pulmonary | 1.36 (1.14, 1.63) | 0.0006 | 1.45 (1.14, 1.85) | 0.0025 |
| Cardiovascular | 1.36 (1.19, 1.56) | <0.0001 | 1.30 (1.06, 1.59) | 0.0103 |
| Infectious | 1.09 (0.86, 1.41) | 0.4790 | 1.03 (0.71, 1.49) | 0.8644 |
| Neurological | 1.92 (1.41, 2.68) | 0.0001 | 2.41 (1.63, 3.61) | <0.0001 |
| Gastrointestinal | 1.58 (1.08, 2.40) | 0.0244 | 1.93 (1.15, 3.27) | 0.0126 |
| Urinary | 1.10 (0.94, 1.29) | 0.2571 | 1.29 (1.02, 1.62) | 0.0334 |
| Surgical | 1.18 (1.08, 1.30) | 0.0005 | 1.35 (1.16, 1.56) | 0.0001 |
| In-hospital mortality | 3.42 (1.42, 11.23) | 0.0169 | 4.83 (1.78, 16.93) | 0.0049 |
| **Perioperative administrative outcomes** | | | | |
| 30-day mortality | 2.21 (1.22, 4.51) | 0.0165 | 2.75 (1.34, 6.10) | 0.0084 |
| Unexpected ICU admission | 1.34 (1.00, 1.85) | 0.0591 | 1.26 (0.84, 1.89) | 0.2666 |
| Readmission within 30 days | 1.07 (0.93, 1.25) | 0.3543 | 1.29 (1.03, 1.61) | 0.0236 |
| Unanticipated surgical approach conversion^a^ | 1.06 (0.92, 1.24) | 0.4220 | 0.99 (0.78, 1.24) | 0.9064 |
| Discharge to home | 0.48 (0.36, 0.62) | <0.0001 | 0.39 (0.27, 0.54) | <0.0001 |
| **Composite Events** | | | | |
| Any post-operative event | 1.22 (1.13, 1.33) | <0.0001 | 1.27 (1.12, 1.45) | 0.0002 |
| Any major complication | 1.24 (1.14, 1.34) | <0.0001 | 1.31 (1.16, 1.49) | <0.0001 |

* Odds ratio relative to ASA = 1 or 2; ASA: American Society of Anesthesiologists; ICU: intensive care unit; ^a^Video assisted thoracic surgery (VATS) to open or robotic to open
