# Supplementary material for: The risk analysis index is an independent predictor of outcomes after lung cancer resection
Source: PLoS One. 2024 May 16;19(5):e0303281. doi: 10.1371/journal.pone.0303281 (PMC11098335; doi:10.1371/journal.pone.0303281)
Supplement: S7 Table — (DOCX) [file pone.0303281.s007.docx]

**S7 Table. Multivariate analysis for CCI and postoperative outcomes**

|  | **Odds ratio* (95% confidence interval)** | | | | | |
| --- | --- | --- | --- | --- | --- | --- |
|  | CCI = 3 | p-value | CCI = 4 | p-value | CCI ≥ 5 | p-value |
| **Postoperative complications** | | | | | | |
| Pulmonary | 1.06 (0.91, 1.23) | 0.4326 | 1.35 (1.09, 1.66) | 0.0055 | 1.48 (1.16, 1.86) | 0.0011 |
| Cardiovascular | 1.25 (1.10, 1.41) | 0.0004 | 1.39 (1.16, 1.66) | 0.0004 | 1.37 (1.12, 1.67) | 0.0022 |
| Infectious | 0.99 (0.78, 1.25) | 0.9173 | 1.22 (0.87, 1.70) | 0.2512 | 1.31 (0.90, 1.88) | 0.1497 |
| Neurological | 1.10 (0.87, 1.38) | 0.4411 | 1.22 (0.88, 1.67) | 0.2201 | 1.77 (1.24, 2.49) | 0.0012 |
| Gastrointestinal | 0.97 (0.69, 1.34) | 0.8349 | 1.67 (1.07, 2.58) | 0.0222 | 1.56 (0.94, 2.50) | 0.0769 |
| Urinary | 1.27 (1.10, 1.48) | 0.0017 | 1.27 (1.02, 1.57) | 0.0335 | 1.40 (1.09, 1.78) | 0.0075 |
| Surgical | 1.08 (0.98, 1.19) | 0.1064 | 1.30 (1.13, 1.50) | 0.0002 | 1.46 (1.25, 1.71) | <0.0001 |
| In-hospital mortality | 1.65 (1.00, 2.70) | 0.0467 | 2.08 (1.06, 3.97) | 0.0288 | 3.23 (1.62, 6.12) | 0.0005 |
| **Perioperative administrative outcomes** | | | | | | |
| 30-day mortality | 1.28 (0.85, 1.91) | 0.2333 | 1.65 (0.95, 2.81) | 0.0680 | 2.63 (1.48, 4.52) | 0.0007 |
| Unexpected ICU admission | 1.11 (0.87, 1.40) | 0.4014 | 1.54 (1.12, 2.12) | 0.0079 | 1.76 (1.24, 2.47) | 0.0013 |
| Readmission within 30 days | 1.13 (0.97, 1.30) | 0.1069 | 1.24 (1.00, 1.53) | 0.0438 | 1.44 (1.14, 1.81) | 0.0020 |
| Unanticipated surgical approach conversion^a^ | 1.06 (0.92, 1.24) | 0.4162 | 1.13 (0.90, 1.40) | 0.2956 | 1.34 (1.05, 1.69) | 0.0173 |
| Discharge to home (n=32,109) | 0.69 (0.57, 0.83) | 0.0001 | 0.59 (0.46, 0.76) | <0.0001 | 0.38 (0.29, 0.49) | <0.0001 |
| **Composite Events** | | | | | | |
| Any post-operative event | 1.18 (1.09, 1.28) | 0.0001 | 1.39 (1.23, 1.57) | <0.0001 | 1.51 (1.32, 1.74) | <0.0001 |
| Any major complication | 1.20 (1.10, 1.30) | <0.0001 | 1.41 (1.25, 1.59) | <0.0001 | 1.56 (1.36, 1.79) | <0.0001 |

* Odds ratio relative to CCI = 2; CCI: Charlson Comorbidity Index; ICU: intensive care unit; ^a^Video assisted thoracic surgery (VATS) to open or robotic to open
