# Supplementary material for: The risk analysis index is an independent predictor of outcomes after lung cancer resection
Source: PLoS One. 2024 May 16;19(5):e0303281. doi: 10.1371/journal.pone.0303281 (PMC11098335; doi:10.1371/journal.pone.0303281)
Supplement: S8 Table — (DOCX) [file pone.0303281.s008.docx]

**S8 Table. Assessment of marginal probability for risk metrics by outcome categories.**

|  | **Marginal probability of events** | | | | | | | |  |
| --- | --- | --- | --- | --- | --- | --- | --- | --- | --- |
| **RAI** | **<35** | **95% CI** | **35-39** | **95% CI** | **40-44** | **95% CI** | **>=45** | **95% CI** | **Monotonicity** |
| **Postoperative complications** | | | | | | |  |  |  |
| Pulmonary | 0.061 | 0.054, 0.068 | 0.071 | 0.066, 0.075 | 0.082 | 0.070, 0.095 | 0.099 | 0.079, 0.119 | Strict |
| Cardiovascular | 0.065 | 0.058, 0.071 | 0.122 | 0.117, 0.128 | 0.120 | 0.105, 0.136 | 0.121 | 0.0978, 0.144 | Partial |
| Infectious | 0.022 | 0.018, 0.026 | 0.027 | 0.024, 0.030 | 0.026 | 0.018, 0.033 | 0.042 | 0.028, 0.057 | Partial |
| Neurological | 0.014 | 0.011, 0.018 | 0.027 | 0.024, 0.030 | 0.039 | 0.030, 0.047 | 0.047 | 0.033, 0.061 | Strict |
| Gastrointestinal | 0.010 | 0.007, 0.012 | 0.014 | 0.012, 0.016 | 0.016 | 0.010, 0.022 | 0.026 | 0.015, 0.038 | Strict |
| Urinary | 0.040 | 0.035, 0.046 | 0.070 | 0.066, 0.075 | 0.097 | 0.083, 0.111 | 0.112 | 0.090, 0.134 | Strict |
| Surgical | 0.190 | 0.179, 0.201 | 0.220 | 0.213, 0.227 | 0.227 | 0.207, 0.247 | 0.245 | 0.214, 0.275 | Strict |
| In-hospital mortality | 0.002 | 0.001, 0.004 | 0.006 | 0.004, 0.007 | 0.005 | 0.002, 0.008 | 0.011 | 0.005, 0.018 | Partial |
| **Perioperative administrative outcomes** | | | | | | |  |  |  |
| 30-day mortality | 0.004 | 0.002, 0.005 | 0.009 | 0.007, 0.010 | 0.012 | 0.008, 0.017 | 0.018 | 0.010, 0.027 | Strict |
| Unexpected ICU admission | 0.060 | 0.047, 0.073 | 0.085 | 0.077, 0.094 | 0.098 | 0.076, 0.120 | 0.084 | 0.056, 0.112 | Partial |
| Readmission within 30 days | 0.062 | 0.056, 0.069 | 0.076 | 0.072, 0.081 | 0.102 | 0.087, 0.117 | 0.098 | 0.076, 0.120 | Partial |
| Unanticipated surgical approach conversion^a^ | 0.067 | 0.060, 0.074 | 0.074 | 0.069, 0.078 | 0.095 | 0.081, 0.109 | 0.073 | 0.055, 0.091 | Partial |
| Discharge to home | 0.974 | 0.970, 0.979 | 0.959 | 0.955, 0.962 | 0.933 | 0.922, 0.944 | 0.884 | 0.863, 0.905 | Strict |
| **Composite Events** | | | | | | |  |  |  |
| Any post-operative event | 0.273 | 0.261, 0.285 | 0.351 | 0.343, 0.359 | 0.365 | 0.342, 0.388 | 0.412 | 0.376, 0.448 | Strict |
| Any major complication | 0.282 | 0.270, 0.294 | 0.363 | 0.355, 0.371 | 0.385 | 0.362, 0.409 | 0.433 | 0.397, 0.470 | Strict |
| **CCI** | **1,2** | **95% CI** | **3** | **95% CI** | **4** | **95% CI** | **≥5** | **95% CI** | **Monotonicity** |
| **Postoperative complications** | | | | | | | | | |
| Pulmonary | 0.065 | 0.060, 0.070 | 0.067 | 0.061, 0.073 | 0.079 | 0.069, 0.088 | 0.084 | 0.073, 0.096 | Strict |
| Cardiovascular | 0.098 | 0.092, 0.104 | 0.114 | 0.106, 0.123 | 0.115 | 0.103, 0.127 | 0.112 | 0.098, 0.126 | Partial |
| Infectious | 0.026 | 0.023, 0.029 | 0.025 | 0.021, 0.029 | 0.029 | 0.023, 0.035 | 0.031 | 0.024, 0.039 | Partial |
| Neurological | 0.024 | 0.020, 0.027 | 0.026 | 0.022, 0.030 | 0.028 | 0.023, 0.034 | 0.041 | 0.032, 0.049 | Strict |
| Gastrointestinal | 0.014 | 0.011, 0.016 | 0.012 | 0.009, 0.015 | 0.018 | 0.013, 0.022 | 0.016 | 0.011, 0.021 | None |
| Urinary | 0.058 | 0.053, 0.063 | 0.073 | 0.066, 0.079 | 0.072 | 0.063, 0.082 | 0.079 | 0.067, 0.091 | Partial |
| Surgical | 0.200 | 0.192, 0.208 | 0.207 | 0.196, 0.218 | 0.226 | 0.211, 0.242 | 0.245 | 0.226, 0.264 | Strict |
| In-hospital mortality | 0.004 | 0.003, 0.005 | 0.006 | 0.004, 0.008 | 0.007 | 0.004, 0.009 | 0.010 | 0.006, 0.014 | Strict |
| **Perioperative administrative outcomes** | | | | | | | | | |
| 30-day mortality | 0.007 | 0.005, 0.009 | 0.008 | 0.006, 0.011 | 0.009 | 0.006, 0.013 | 0.015 | 0.010, 0.020 | Strict |
| Unexpected ICU admission | 0.075 | 0.065, 0.085 | 0.078 | 0.067, 0.090 | 0.095 | 0.077, 0.126 | 0.104 | 0.083, 0.126 | Strict |
| Readmission within 30 days | 0.072 | 0.067, 0.078 | 0.079 | 0.072, 0.087 | 0.084 | 0.073, 0.094 | 0.095 | 0.082, 0.109 | Strict |
| Unanticipated surgical approach conversion^a^ | 0.071 | 0.066, 0.077 | 0.072 | 0.065, 0.079 | 0.069 | 0.060, 0.088 | 0.069 | 0.060, 0.079 | Strict |
| Discharge to home | 0.967 | 0.964, 0.971 | 0.955 | 0.950, 0.960 | 0.951 | 0.944, 0.959 | 0.928 | 0.917, 0.939 | Strict |
| **Composite Events** | | | | | | | | | |
| Any post-operative event | 0.312 | 0303, 0.322 | 0.339 | 0.327, 0.352 | 0.358 | 0.341, 0.376 | 0.375 | 0.354, 0.396 | Strict |
| Any major complication | 0.322 | 0.312, 0.331 | 0.351 | 0.339, 0.364 | 0.367 | 0.349, 0.385 | 0.388 | 0.370, 0.400 | Strict |
| **ASA** | **1,2** | **95% CI** | **3** | **95% CI** | **4,5,6** | **95% CI** |  |  | **Monotonicity** |
| **Postoperative complications** | | | | | | | | | |
| Pulmonary | 0.054 | 0.045, 0.062 | 0.071 | 0.068, 0.075 | 0.075 | 0.064, 0.087 |  |  | Strict |
| Cardiovascular | 0.083 | 0.074, 0.093 | 0.110 | 0.105, 0.114 | 0.105 | 0.091, 0.119 |  |  | Partial |
| Infectious | 0.023 | 0.019, 0.030 | 0.027 | 0.025, 0.029 | 0.026 | 0.019, 0.033 |  |  | Partial |
| Neurological | 0.015 | 0.010, 0.019 | 0.028 | 0.026, 0.030 | 0.035 | 0.027, 0.043 |  |  | Strict |
| Gastrointestinal | 0.009 | 0.006, 0.013 | 0.014 | 0.013, 0.016 | 0.017 | 0.012, 0.023 |  |  | Strict |
| Urinary | 0.060 | 0.052, 0.069 | 0.066 | 0.063, 0.069 | 0.077 | 0.065, 0.089 |  |  | Strict |
| Surgical | 0.185 | 0.173, 0.198 | 0.211 | 0.206, 0.217 | 0.233 | 0.213, 0.253 |  |  | Strict |
| In-hospital mortality | 0.002 | <0.001, 0.003 | 0.006 | 0.005, 0.007 | 0.008 | 0.005, 0.012 |  |  | Strict |
| **Perioperative administrative outcomes** | | | | | | | | | |
| 30-day mortality | 0.004 | 0.002, 0.007 | 0.009 | 0.008, 0.010 | 0.011 | 0.007, 0.015 |  |  | Strict |
| Unexpected ICU admission | 0.065 | 0.047, 0.083 | 0.085 | 0.079, 0.092 | 0.080 | 0.061, 0.100 |  |  | Partial |
| Readmission within 30 days | 0.073 | 0.063, 0.082 | 0.078 | 0.074, 0.081 | 0.092 | 0.078, 0.105 |  |  | Partial |
| Unanticipated surgical approach conversion^a^ | 0.069 | 0.060, 0.078 | 0.073 | 0.070, 0.077 | 0.068 | 0.057, 0.080 |  |  | Partial |
| Discharge to home | 0.978 | 0.972, 0.984 | 0.956 | 0.953, 0.959 | 0.946 | 0.937, 0.956 |  |  | Strict |
| **Composite Events** | | | | | | | | | |
| Any post-operative event | 0.294 | 0.279, 0.309 | 0.336 | 0.330, 0.343 | 0.345 | 0.323, 0.367 |  |  | Strict |
| Any major complication | 0.302 | 0.286, 0.317 | 0.347 | 0.340, 0.353 | 0.360 | 0.337, 0.382 |  |  | Strict |

RAI: Risk Analysis Index; CCI: Charlson comorbidity index; ASA: American Society of Anesthesiologists
