# Supplementary figures and images for: The risk analysis index is an independent predictor of outcomes after lung cancer resection
Source: PLoS One. 2024 May 16;19(5):e0303281. doi: 10.1371/journal.pone.0303281 (PMC11098335; doi:10.1371/journal.pone.0303281)

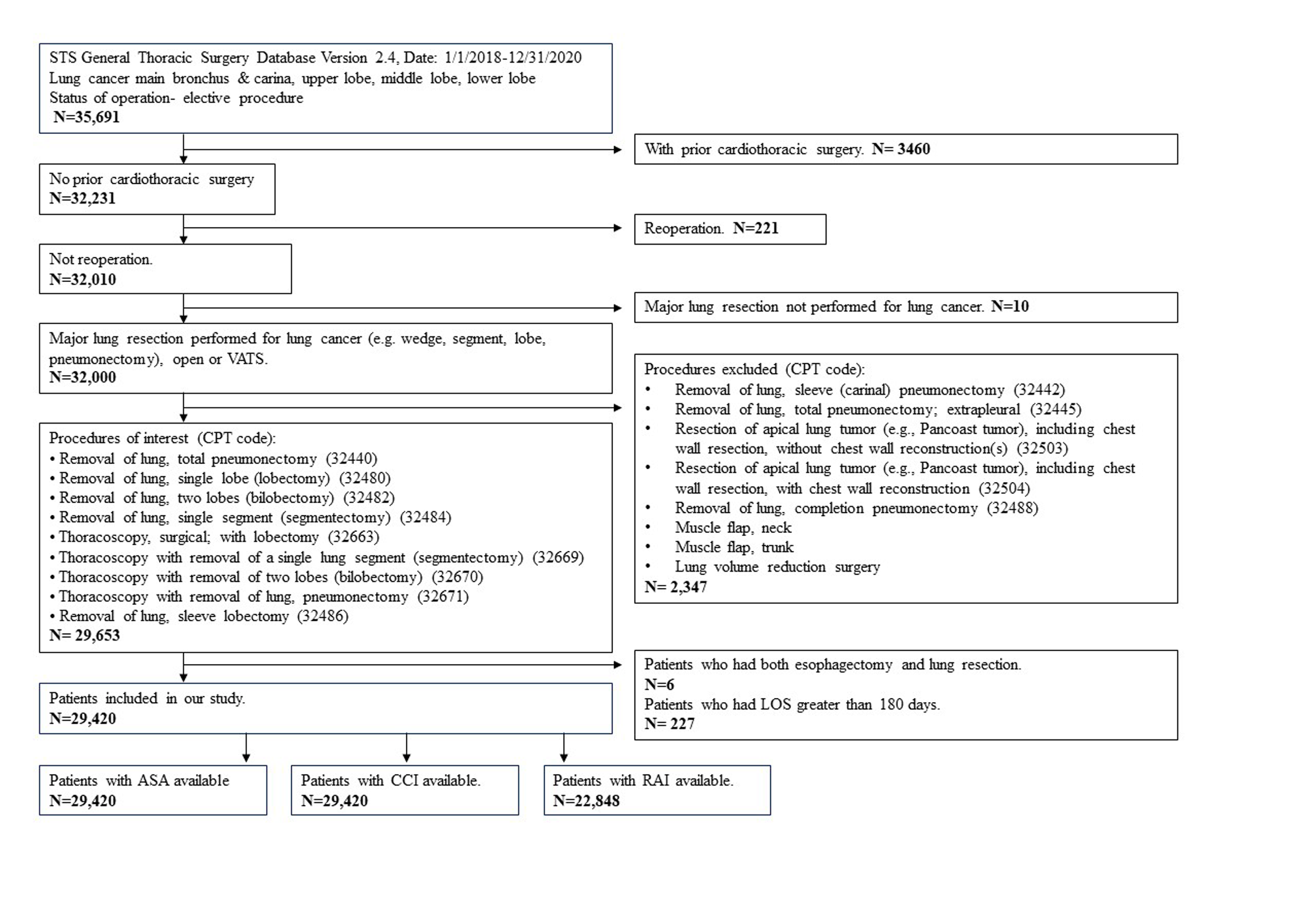

Supplement: S1 Fig — (TIF) [file pone.0303281.s009.tif]
