# Supplementary material for: The risk analysis index is an independent predictor of outcomes after lung cancer resection
Source: PLoS One. 2024 May 16;19(5):e0303281. doi: 10.1371/journal.pone.0303281 (PMC11098335; doi:10.1371/journal.pone.0303281)
Supplement: S1 Text — (DOCX) [file pone.0303281.s010.docx]

S1 Text

Data for this study were obtained from a third party, the Society of Thoracic Surgeons. The data use agreement specifically prohibits sharing of the data in any manner.

Application for access to data used in this study may be submitted to the Society of Thoracic Surgeons at <https://www.sts.org/sts-national-database>.

The specific request format we used is outlined below:

**Inclusion criteria**

- Lung cancer main bronchus & carina, upper lobe, middle lobe, lower lobe **(V2.41 1250-1270 [150, 160, 170, 180])**
- Operations for lung cancer **(V2.41 1580-1590)**
- Status of operation- elective procedure **(V2.41 1400 [3])**
- Robotic Technology Assisted (both yes and no) **(V2.41 1420)**
- Primary procedure performed: Lung Resection

Extent of lung resection (segmentectomy, lobectomy, bilobectomy, or pneumonectomy)

Approach (open; minimally invasive (VATS or robotic))

**V2.41 1490** Primary procedure

- - - **2470 Removal of lung, total pneumonectomy; (32440)**
    - **2500 Removal of lung, single lobe (lobectomy) (32480)**
    - **2510 Removal of lung, two lobes (bilobectomy) (32482)**
    - **2520 Removal of lung, single segment (segmentectomy) (32484)**
    - **2530 Removal of lung, sleeve lobectomy (32486)**
    - **2540 Removal of lung, completion pneumonectomy (32488)**
    - **2800 Thoracoscopy, surgical; with lobectomy (32663)**
    - **4100 Thoracoscopy with removal of a single lung segment (segmentectomy) (32669)**
    - **4110 Thoracoscopy with removal of two lobes (bilobectomy) (32670)**
    - **4120 Thoracoscopy with removal of lung, pneumonectomy (32671)**

**Exclusion criteria**

- Final diagnosis other than primary lung cancer
- Status of operation- Urgent procedure, Emergent operation, Palliative **(V2.41 1400 [1,2,4])**
- Primary procedure performed

Extent of lung resection (wedge, extra-pleural or sleeve pneumonectomy)

**V2.41 1490** Primary procedure

- - - **2480 Removal of lung, sleeve (carinal) pneumonectomy (32442)**
    - **2490 Removal of lung, total pneumonectomy; extrapleural (32445)**
    - **4070 Thoracoscopy with therapeutic wedge resection (eg mass or nodule, initial, unilateral (32666)**
    - **4080 Thoracoscopy with therapeutic wedge resection (eg mass or nodule), each additional resection, ipsilateral (32667)**
    - **2580 Resection of apical lung tumor (e.g., Pancoast tumor), including chest wall resection, without chest wall reconstruction(s) (32503)**
    - **4140 Thoracotomy with therapeutic wedge resection (eg mass nodule) initial (32505)**
    - **2590 Resection of apical lung tumor (e.g., Pancoast tumor), including chest wall resection, with chest wall reconstruction (32504)**
    - **4150 Thoracotomy with therapeutic wedge resection (eg mass nodule) each additional resection, ipsilateral (+32506), List separately in addition to primary proc code**
    - **2480 Removal of lung, sleeve (carinal) pneumonectomy (32442)**

**RAI Variables**

- Age **(V2.41 170)**
- Gender (1,2) **(V2.41 190)**
- Living Status (1-4) **(V2.41 1040)**
- Shortness of breath (primary or secondary diagnosis) **(V2.41 1260-1300; if [1660] then patient considered to have shortness of breath)**
- Weight loss over past 3 months (yes/no) **(V2.41 430)**
- Poor appetite (yes/no) **(V2.41 430)**
- Congestive heart failure (**yes**/no) **(V2.41 450)**
- Renal Dysfunction: Dialysis (**yes**/no) **(V2.41 680)**
- Cancer: any of the following: Lung cancer main bronchus & carina, upper lobe, middle lobe, lower lobe **(V2.41 1250-1270 [150, 160, 170, 180]);** Coexisting cancer (**yes**/no) **(V2.41 690)**
- Recent cognitive decline: Dementia Neuro Dysfunction (**yes**/no) **(V2.41 1020)**
- Functional status (1,2,3) (4- unknown) **(V2.41 1050)**

**Independent Variables**

- Date of surgery **(V2.41 1310)**

**Demographics**

- Race/ethnicity **(V2.41 200-270)**

**Preoperative Evaluations**

- ASA classification (1-6) **(V2.41 1480)**
- Body Mass Index (height and weight available for all)
  - Ht (cm) **(V2.41 410)**
  - Wt (kg) **(V2.41 420)**
- % predicted FEV1 (FEV1%) **(V2.41 930)**
- % predicted DLCO (DLCO%) **(V2.41 960)**
- Pneumonia (secondary diagnosis) **(V2.41 1280-1300 [280])**
- Cigarette use: ([never OR past (stop > 1 mo.)], current) **(V2.41 970)**
- Pack years (packyear) **(V2.41 980-990)**
- Pulmonary hypertension (yes/no) **(V2.41 550)**
- Interstitial fibrosis (yes/no) **(V2.41 560)**
- Hypertension (yes/no) **(V2.41 440)**
- Ecog (0-5) **(V2.41 1070)**
- Ejection Fraction (1-99) **(V2.41 460)**
- Coronary artery disease (yes/no) **(V2.41 470)**
- Previous MI (yes/no) **(V2.41 480)**
- Afib (yes/no) **(V2.41 490)**
- Valvular heart disease (yes/no) **(V2.41 500)**
- Diabetes: (use yes/no) **(V2.41 650)**
- MVD (yes/no) **(V2.41 580)**
- Cerebrovascular disease (CerebroHx): (use yes/no) **(V2.41 610)**
- Permanent neuro impairment (yes/no) **(V2.41 620)**
- Liver Dysfunction (yes/mo) **(V2.41 670)**
- Renal Dysfunction: Dialysis (yes/no) **(V2.41 680)** or Last Creatinine level (> 2 or <2) **(V2.41 880)**
- Induction radiation use only within 6 months, same disease (**yes**/no), (**1**,2,3,4) **(V2.41 720-730)**
- Induction chemotherapy therapy use only within 6 months, same disease (yes/no), (1,2,3,4) **(V2.41 700-710)**
- Clinical

Lung Cancer

- - cT **(V2.41 1880)**
  - cN **(V2.41 1890)**
  - cM **(V2.41 1900**)
- Prior cardiothoracic surgery (yes/no) **(V2.41 750-810)**
- Reoperation (yes/no) **(V2.41 1410)**

**Operative Details**

- Conversion MIS to open (1. VATS to open, 2. Robot to VATs, 3. Robot to open, 4. No) **(V2.41 1430)**
- Procedure time (start to finish)
  - Procedure start time **(V2.41 1360)**
- Procedure end time **(V2.41 1370)**
- Pathological stage

Lung Cancer

- pT  **(V2.41 1920)**
- pN **(V2.41 1930)**
- PM **(V2.41 1940)**

**ICU LOS**

- Intensive care unit days initial ICU + additional ICU (yes/no, days, yes/no, days) **(V2.41 3270-3300)**
- Unexpected admission to ICU (yes/no) **(V2.41 3840)**

**Hospital length of stay**

- Date of surgery **(V2.41 1310)**
- Date of discharge **(V2.41 3870)**

**Discharge Location**

Discharge Location (1 Home, 2 Extended Care/Transitional Care Unit/Rehab. 3 Other Hospital. 4 Nursing Home 5 Hospice. 777 Other) **(V2.41 3890)**

**Readmission**

- Readmission with 30 days (yes/no) **(V2.41 3930)**

**Complications**

- **Pulmonary events**
  - Atelectasis requiring bronchoscopy (yes/no) **(V2.41 3440)**
  - Pneumonia (yes/no) **(V2.41 3460)**
  - ARDS (yes/no) **(V2.41 3470)**
  - Ventilator support > 48 hrs (yes/no) **(V2.41 3520)**
  - Tracheostomy (yes/no) **(V2.41 3530)**
  - Respiratory Failure (yes/no) **(V2.41 3480)**
  - Other (yes/no) **(V2.41 3550)**
- **Cardiovascular events**
  - Deep venous thrombosis (yes/no) **(V2.41 3590)**
  - Atrial arrhythmia requiring treatment (yes/no) **(V2.41 3560)**
  - Ventricular arrhythmia requiring treatment (yes/no) **(V2.41 3570)**
  - Myocardial infarction (yes/no) **(V2.41 3580)**
  - Other CV event (yes/no) **(V2.41 3600)**
  - Pulmonary embolus (yes/no) **(V2.41 3500)**
- **Infection**
  - Empyema (yes/no) **(V2.41 3730)**
  - Sepsis (yes/no) **(V2.41 3750)**
  - Surgical Site infection (1 None, 2 Superficial, 3 Deep,4 Organ space (intra-abdominal abscess, mediastinitis)) **(V2.41 3740)**
  - Other infection requiring IV antibiotics (yes/no) **(V2.41 3760)**
- **Neurological**
  - Central neurologic event (yes/no) **(V2.41 3770)**
  - Other neurological Event (yes/no) **(V2.41 3800)**
  - Delirium (yes/no) **(V2.41 3790)**
- **Urinary**
  - New renal failure or worsening Cr ≥ 3X preop (yes/no) **(V2.41 3810)**
  - UTI (yes/no) **(V2.41 3700)**
  - Urinary retention req. Catheterization (yes/no) **(V2.41 3710)**
  - Discharged With Foley Catheter (yes/no) **(V2.41 3720)**
- **Surgical**
  - Air leak > 5 days duration **(V2.41 3430)**
  - Intraop or periop blood transfusion: yes, no **(Intra-op: V2.41 1460)**
  - Post-op blood transfusion **(V2.41 3680)**
  - Chylothorax (yes/no) **(V2.41 3390)**
  - Chylothorax requiring intervention (yes/no) **(V2.41 3400-3410)**
  - Chylothorax requiring medical therapy (yes/no) **(V2.41 3820)**
  - Pneumothorax requiring CT (yes/no) **(V2.41 3510)**
  - Pleural effusion requiring drainage (yes/no) **(V2.41 3450)**
  - Unanticipated PostOP Invasive Procedure (yes/no) **(V2.41 3330)**
  - Primary Reason for procedure **(V2.41 3340 [1 Bleeding,3 Bronchopleural Fistula,4 Empyema,8 Middle lobectomy for torsion, 7 Conduit necrosis/failure following esophageal surgery, 6 Other])**
  - Other events requiring OR with GA (yes, no) (**V2.41 3830)**
  - Bronchopleural fistula (yes,no) **(V2.41 3490)**
  - Discharged with chest tube (yes/no) **(V2.41 3900)**

**Mortality**

- - In Hospital **mortality** (Alive, dead) **(V2.41 3880)**
  - 30 days **mortality** (1 Alive,**2 Dead**,3 Unknown) **(V2.41 3950)**

**Postoperative Event**

- **Any postoperative event (yes/no)** **(V2.41 3310)**
- **Any major postoperative event: (yes/no) (Any pulmonary OR cardiovascular OR other OR GI OR Neuro OR mortality)**
